# Dynamic Group Multi-party Quantum Key Agreement

**DOI:** 10.1038/s41598-018-21658-6

**Published:** 2018-03-15

**Authors:** Yao-Hsin Chou, Guo-Jyun Zeng, Zhe-Hua Chang, Shu-Yu Kuo

**Affiliations:** Department of Computer Science and Information Engineering, National Chi Nan University, Puli, 54561 Taiwan

## Abstract

This paper presents a novel dynamic group multi-party Quantum Key Agreement (DGMQKA) protocol, achieved by a multicast transmission method. The proposed method is able to achieve arbitrary number of groups and members under the same resources. In addition, it can be dynamically adjusted by joining a new member, combining two groups into one group, revoking an old member and dividing one group into two groups, for different and complex situations. Furthermore, the proposed protocol can be of help to research into Quantum Secret Sharing (QSS), which it complements. The security analysis shows that the proposed protocol can resist both external and internal attacks. In consumption comparison, the proposed protocol using the multicast transmission method is more effective than other current MQKA protocols.

## Introduction

In 1984, Bennett and Brassard^1^ proposed the first quantum key distribution (QKD, also called BB84) protocol. Its security is based on the quantum physics and guarantees the unconditional security^2–6^, not only theoretically but also in actual implementation. Furthermore, the superposition and entanglement properties enable researchers to develop the quantum algorithm required to crack the famous RSA cryptosystem by quantum parallel computing^7–9^. A quantum algorithm can be a potent weapon to threaten classical cryptography. It enables researchers to develop quantum cryptography, which offers security based on physical laws rather than computational complexity, to defend against attacks from quantum computers. In addition, other interesting applications differing from the past are developed, such as quantum dense coding^10^ and teleportation^11^. So far, three interesting branches of quantum cryptography are Quantum Key Distribution (QKD), Quantum Secure Direct Communication (QSDC), and Quantum Secret Sharing (QSS).

QKD: After BB84, in 1991, Ekert proposed the first QKD protocol (also called E91) based on EPR pairs. Then, Bennett *et al*.^12^ proved that E91 is equivalent to BB84^1^. Bennett^13^ later proposed using non-orthogonal bases and two qubit states to implement QKD in 1992. In addition, other researchers developed some new protocols for enhancing the performance of QKD protocol: Lo *et al*.^14^ enhanced the key rate, Deng and Long^15^ improved the key usage rate by controlling the order rearrangement technique; they^16^ also saved the storage space by bidirectional QKD in 2004. However, a QKD protocol is not only a protocol design; it also needs to consider implementation. Therefore, more and more researchers have discussed the implementation issue, such as imperfect single-qubit sources^17^, noise channel^18^, and imperfect device^6,19,20^.

QSDC: Furthermore, another novel branch of quantum cryptography, which allows the agents can transmit secret messages by using quantum resources directly. Long and Liu^21^. proposed first QSDC protocol by using entangled qubits with twice transmissions and full transmission performance in February 2002. Then Boström and Felbinger^22^ proposed famous QSDC protocol nowadays, called ping-pong protocol in October 2002. Nguyen^23^ designed a QSDC protocol, it allowed two agents exchange their secret message at once transmission, in 2004, called quantum dialogue or bidirectional QSDC (BQSDC) protocol. Gao *et al*.^24^ proposed a controlled QSDC (CQSDC) protocol based on quantum teleportation^11^, in 2005, which adds a controller to help the receiver to decrypt the secret message but he cannot know anything about the message. The role of a controller can be mapped as a telecommunications company who should provide the service but should not monitor the transmission. Furthermore, Jin *et al*.^25^ proposed a multi-party QSDC (MQSDC) protocol, which allows all agents to exchange their secret message, in 2006, simultaneously. In the same year, Man and Xia^26^ combined features of BQSDC and CQSDC to design controlled bidirectional QSDC (CBQSDC) protocol. On the other hand, another group of researchers also focused on the implementation such as the development of QKD. Long and Liu^27,28^ proposed two QSDC protocol based on entangled state and single-qubit, respectively, and they are feasible with the present-day technique in 2003. Most recently, Zhang *et al*.^29^ implemented Long’s^21,27^ QSDC protocol with quantum memory to achieve approximately 90% for entanglement decoding for the experiment.

QSS: It needs all or some agents to decode the secret message by cooperating. In 1999, Hillery *et al*.^30^ proposed a first QSS concept, also called HBB99, which shares a qubit between all agents using teleportation based on GHZ states. Later, Cleve *et al*.^31^ proposed a (*k*, *n*) threshold QSS protocol based on quantum error correction code to share quantum information, and proved that the threshold must be $k\le n < 2k-1$, corresponding to a no-cloning theorem^32^, where *k* is the threshold and *n* is number of total agents. In 2003, Guo^33^ proposed a QSS scheme without entanglement and Hsu^34^ proposed a QSS scheme based on Grover’s algorithm. Xiao *et al*.^35^ generalized HBB99 into arbitrary multi parties in 2004. Furthermore, Zhang *et al*.^36,37^, Deng *et al*.^38^, Hwang *et al*.^39^, and Chou *et al*.^40^ all proposed efficient multi party QSS (MQSS) schemes during the period of 2005 to 2012. After that, Jia *et al*.^41^, Hsu *et al*.^42^, Liao *et al*.^43^, and Liu *et al*.^44^ also proposed the scheme of dynamic MQSDC (DMQSDC) during the period of 2012 to 2016. On the other hand, Lance *et al*.^45^ tried to implement the threshold QSS scheme^31,46^, and demonstrated (2, 3) threshold quantum secret sharing in a tripartite entangled state.

The above subtopics of quantum cryptography were developed diversely and widely used in modern application^47^; however, the development of the Quantum Key Agreement (QKA), which is also an important subset of QKD, was delayed until 2004. The rule of QKA is stricter than QKD. The key generation in QKD is prepared by one participant, and then distributed to the others. However, there is an important condition in QKA: the key must be determined by all participants together, rather than by one participant individually, i.e. every participant can change the key, but cannot determine the key. The purpose of QKA is to gather all the pieces in each participant’s hand to create a secret key, while QSS^30,35,36,41–44^ aims to divide a secret key into many pieces and distribute one piece to each participant. The concept of QKA is analogous to the reverse procedure of QSS, so both complement each other through the reduction from MQSS to MQSDC and then to multi party QKA. Not all MQSS protocols can be mapped easily, for example, Zhang and Man’s QSS protocol removes the sender Alice; the situation of the remaining agents, Bob and Charlie, can be considered as quantum dialogue^23^; they can then exchange their secret (a part of key) simultaneously. In the key exchanged case, the final key can be generated from their secret, and this situation is key agreement.

The first QKA protocol based on the quantum teleportation^11^ technique was proposed by Zhou *et al*.^48^ in 2004. Soon after, Hsueh *et al*.^49^ proposed another QKA protocol with maximally entangled states in the same year. Afterwards, in 2009, Tsai *et al*.^50^ observed that Zhou *et al*.’s protocol is vulnerable to a participant attack, in which one participant is able to determine the key alone, and they also presented an improvement to the protocol. In 2010, Chong *et al*.^51^ demonstrated that Zhou *et al*. and Tsai *et al*.’s protocols were not fair, because in both, the key is generated by random measurement result, and not by the opinion of participants. They proposed a QKA protocol based on BB84, in which the key is formed by consent of all participants. After that, Chong *et al*.^52^ pointed out security vulnerabilities in Hsueh *et al*.’s protocol^49^, and presented an improvement to avoid attacks exploiting those vulnerabilities. However, it is the opinion of this study that Zhou *et al*.’s protocol is infeasible because it operates contrary to the no-cloning theorem^32^. Due to the strict restrictions of key generation, the QKA protocols^48–52^ proposed only considered two-party interactions, but failed to consider multi-party ones.

The concept of multi-party Quantum Key Agreement (MQKA) was first introduced in 2012 when Shi *et al*.^53^ proposed the first MQKA protocol using Bell states and Bell measurement. However, Liu *et al*.^54^ pointed out the flaws in this protocol, and then proposed another MQKA protocol using single particles. Since then, many more MQKA protocols^55–60^ have been proposed. In order to achieve the key generation conditions, and the extension from two-party to multi-party QKA, these MQKA protocols^53–60^ used the unicast transmission method, exchanging information on one for one. In this way, the resource consumption will increase rapidly with the increase of participants. In 2016, Zeng *et al*.^61^ proposed an efficient MQKA protocol based on MQSDC^25^ using ‘broadcast’ transmission, which means all of agents can exchange their secret message, not only further improving efficiency, but also economizing time and quantum resource.

Although more and more QKA protocols are being proposed, a protocol with dynamic properties has not yet been presented. Real-world situations are very complex and must be adjusted to dynamically. In common QKA protocols, the quantum resources are always transmitted during key configuration. However, this may not be effective for more complex situations. Take wireless sensor networks, for example. All sensors in these networks are randomly distributed in any scenario. It is unknown which sensors will form a group later. As a result, it would be very convenient if all sensors were initially under the same resource distribution. During the sensing period, some sensors may need to combine with or separate from groups, and other new members may need to join groups. This will result in very heavy loading for common QKA protocols.

This paper therefore proposes a dynamic group MQKA based on previous studies (MQSDC^25,61^ and DMQSS^41–44^). That is, any participant can join or leave the group, and any two groups can combine into one group easily. Besides, under the same resource distribution, this protocol can make the corresponding variation according to different grouping demands. This means that proposed protocol is applicable to arbitrary grouping combination. This dynamic group MQKA protocol is more flexible for establishing groups and generating group keys, and more practical for matching the demand in real life. This is because these two processes complement each other. It will also be helpful for future QSS research.

## The proposed method

The basic idea of the proposed protocol is an improvement from the work of Zeng *et al*.^61^. The broadcast transmission method makes Zeng *et al*.’s protocol very efficient. Similarly, through broadcast transmission, the proposed protocol is designed to group property that can be adjusted dynamically and makes different group can only obtain their own group key which called multicast in the proposed protocol. The proposed protocol is able to create arbitrary groups using the same quantum resources. As long as all members configure the same initial quantum resources, it is impossible to know how many groups there will be and how many members will be in each group. In addition, because the proposed protocol is just a cryptosystem, it focuses on key configuration and whether all members put the correct key intentions into the protocol is not discussed.

The first example is in the easiest case, a two-group DGMQKA, where two groups generate respective groups and group key using the same entangled state. The second example is a multi-group case, multi-group DGMQKA, which is that more arbitrary number of groups and members perform the group key agreement, and is generalized from the two-group case. The third example is key generation, which shows every group how to exact their respective group key. The final example is the dynamic of the proposed protocol, in which any participant can join or leave a group, and any two groups can combine into one group, or any one group can divide into two groups.

Because of the flexibility of dynamic and group, this DGMQKA protocol is very suitable for complex scenarios, such as the scenario of wireless sensor network. The proposed protocol can satisfy any different and complex situations through joining a new sensor, revoking an old sensor, combining two groups of sensors into one group and dividing one group of sensors into two groups. In the scenario of wireless sensor network, there are many sensors in groups, and they need the group key to communicate with each group member. They will deliver information through other group sensors. For avoiding being eavesdropped, they can perform group key agreement. The group key can be used to encrypt and decrypt the information. Besides, if the sensors are movable, the situations of groups and members will be extremely variable. Therefore, this study can be very useful for any scenario which needs flexibility. The previous studies is only for single group without flexibility, and this study is the first MQKA protocol with properties of group and dynamic. In addition, quantum secret sharing is to divide a message or key into many pieces, while quantum key agreement is to combine many pieces of messages or keys into one. Because the two research areas complement each other, this study is also helpful for the researches of quantum secret sharing.

### The proposed two-group DGMQKA protocol

The simplest case is two groups including 2 (the smallest even number, greater than 1) and 3 (the smallest odd number, greater than 1) participants. Because the encryption and decryption methods will differ with the even or odd number of participants in Zeng *et al*.’s protocol^61^, the smallest cases of even and odd numbers will be discussed here. Suppose that there are five member, *G*_1_ (the subscript is the group number) is composed of two members, ${M}_{1}^{1}$ and ${M}_{2}^{1}$ (the upper superscript is the group number and the lower subscript is the member number), and *G*_2_ is composed of three members: ${M}_{3}^{2}$, ${M}_{4}^{2}$ and ${M}_{5}^{2}$. The members of the two groups will respectively generate a specific group key through operation information that each performs and the measurement result that the group leader announces, but cannot be known by any other group. The protocol is composed of five steps, as follows:

1. Initially, there is a member who prepares the quantum resources, and this member can be any member of the group. Here, suppose that ${M}_{1}^{1}$ prepares an ordered sequence composed of *N* five-qubit entangled states^21^: $\frac{1}{\sqrt{2}}{(|00000\rangle +|11111\rangle )}_{12345}$. ${M}_{1}^{1}$ takes each qubit *i*(*i* = 1, 2, …, 5) in *N* five-qubits entangled states to form five ordered sequences, *S*_*i*_(*i* = 1, 2, …, 5) (the subscript is the sequence index). ${M}_{1}^{1}$ randomly inserts detection qubits^62^ in one of four states $\{|0\rangle ,|1\rangle ,|+\rangle ,|-\rangle \}$ randomly into *S*_2_, *S*_3_, *S*_4_ and *S*_5_ for channel checking. After this, ${M}_{1}^{1}$ sends *S*_2_, *S*_3_, *S*_4_ and *S*_5_ with detection qubits^62^ to ${M}_{2}^{1}$, ${M}_{3}^{2}$, ${M}_{4}^{2}$ and ${M}_{5}^{2}$, respectively.
2. After ${M}_{2}^{1}$, ${M}_{3}^{2}$, ${M}_{4}^{2}$ and ${M}_{5}^{2}$ receive *S*_2_, *S*_3_, *S*_4_ and *S*_5_ with detection qubits^62^, ${M}_{1}^{1}$ announces the positions and states of the detection qubits^62^ to ${M}_{2}^{1}$, ${M}_{3}^{2}$, ${M}_{4}^{2}$ and ${M}_{5}^{2}$. Then ${M}_{2}^{1}$, ${M}_{3}^{2}$, ${M}_{4}^{2}$ and ${M}_{5}^{2}$ use the corresponding bases to measure these detection qubits^62^, and check the states of these detection qubits^62^. If the error rate is higher than the threshold, they will abort this communication; otherwise, they go to the next step.
3. Then, every member will be the group leader, in turn, who measures the entangled states and announces the measurement results. Hence, the order of the group leader in *G*_1_ is ${M}_{1}^{1}$, ${M}_{2}^{1}$, ${M}_{1}^{1}$, …, ${M}_{2}^{1}$ and the order of the group leader in *G*_2_ is ${M}_{3}^{2}$, ${M}_{4}^{2}$, ${M}_{5}^{2}$, …, ${M}_{5}^{2}$; here, ${M}_{1}^{1}$ and ${M}_{3}^{2}$ are the group leaders of *G*_1_ and *G*_2_, respectively. Next, both *G*_1_ and *G*_2_ need to calculate the number of entangled qubits; they have to be $\lceil \frac{P+1}{2}\rceil +\lceil \frac{P-1}{2}\rceil$ with the number of group members *P*. The reason will be explained in the key generation section. Here, the number of members in *G*_1_ is 2, so the number of entangled qubits has to be 3. Therefore, group leader ${M}_{1}^{1}$ in *G*_1_ prepares an additional sequence which is composed of *N* single qubits in ${|0\rangle }_{1^{\prime} }$. Then, ${M}_{1}^{1}$ performs the *CNOT*_11′_ operation on *S*_1_ and *S*_1′_, where each qubit in *S*_1_ is a control bit and each corresponding qubit in *S*_1′_ is a target bit. Every five-qubit entangled state $\frac{1}{\sqrt{2}}{(|00000\rangle +|11111\rangle )}_{12345}$ will be turned into six-qubit entangled state: $\frac{1}{\sqrt{2}}{(|000000\rangle +|111111\rangle )}_{11^{\prime} 2345}$. Similarly, the number of members in *G*_2_ is 3, so the number of entangled qubits has to be 3 (Fig. 1). After all the groups have gone through the above steps, all the members perform one of two operations {*I*, *X*} based on their own choice for a key that represents “0” or “1” on the qubits sequences on hand, as shown in Fig. 1. Then, every member will be the group leader in turn who measures the entangled states and announces the measurement results. Hence, the order of group leader in *G*_1_ is ${M}_{1}^{1}$, ${M}_{2}^{1}$, ${M}_{1}^{1}$, …, ${M}_{2}^{1}$ and the order of group leader in *G*_2_ is ${M}_{3}^{2}$, ${M}_{4}^{2}$, ${M}_{5}^{2}$, …, ${M}_{5}^{2}$.

**Figure 1:**
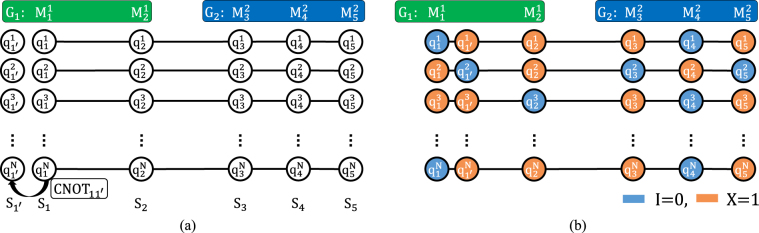
The procedure of two-group DGMQKA step 3. (**a**) The process of odd group, (**b**) The qubit states after operations.
4. After the key encryption, all members randomly insert the detection qubits^62^ for channel checking as ${M}_{1}^{1}$ did in step 1. They send their qubits sequences to their group leader. When every group leader receives the qubit sequences with detection qubits^62^ from members, they announce the positions and states of the detection qubits^62^ for the channel checking, and check the error rate. They abort this communication if the error rate is higher than the threshold; otherwise, they go to the next step.
5. All group leaders perform the GHZ measurement and announce the measurement results. According to Table 1, every member can distinguish the operations of each member of their group by the measurement result that their group leader announced and own operation information done on the qubit, but not of members belonging to other groups. For example, if the measurement results announced by group leader ${M}_{1}^{1}$ and ${M}_{3}^{2}$ are ${|{{\rm{\psi }}}_{000}\rangle }_{11^{\prime} 2}$ and ${|{{\rm{\psi }}}_{000}\rangle }_{345}$, it means that the possible operation combinations for *G*_1_ are *III*_11′2_ or *XXX*_11′2_ and the possible operation combinations of *G*_2_ are *III*_345_ or *XXX*_345_, respectively, as shown in Fig. 2. For *G*_1_, ${M}_{1}^{1}$ can know that ${M}_{2}^{1}$’s operation is *I*_2_ through its own operations *I*_1_, *I*_1′_ and the measurement result of *G*_1_. ${M}_{2}^{1}$ can also know that ${M}_{1}^{1}$’s operation is *I*_1_ and *I*_1′_ with its own operation *I*_2_ and measurement result of *G*_1_. However, they are unable to identify the operations combination of *G*_2_, which are *III*_345_ or *XXX*_345_, because they cannot get operation information from the members of the *G*_1_. Similarly, for *G*_2_, the members of *G*_2_ can only know the operations performed by other members in *G*_2_, but they cannot know the operations of the *G*_1_, which are *III*_11′2_ or *XXX*_11′2_. Finally, the rule of key exaction is discussed in the following section.

**Table 1:** Measurement result of two-groups DGMQKA.

*G*2	*III*_345_ 0	*XXX*_345_ 1	*IIX*_345_ 1	*XXI*_345_ 0	*IXI*_345_ 1	*XIX*_345_ 0	*IX*_345_ 0	*XII*_345_ 1
*G*1								
*III*11′2 0	${|{{\rm{\Psi }}}_{000}\rangle }_{11^{\prime} 2}{|{{\rm{\Psi }}}_{000}\rangle }_{345}$ or ${|{{\rm{\Psi }}}_{100}\rangle }_{11^{\prime} 2}{|{{\rm{\Psi }}}_{100}\rangle }_{345}$		${|{{\rm{\Psi }}}_{000}\rangle }_{11^{\prime} 2}{|{{\rm{\Psi }}}_{001}\rangle }_{345}$ or ${|{{\rm{\Psi }}}_{100}\rangle }_{11^{\prime} 2}{|{{\rm{\Psi }}}_{101}\rangle }_{345}$		${|{{\rm{\Psi }}}_{000}\rangle }_{11^{\prime} 2}{|{{\rm{\Psi }}}_{001}\rangle }_{345}$ or ${|{{\rm{\Psi }}}_{100}\rangle }_{11^{\prime} 2}{|{{\rm{\Psi }}}_{110}\rangle }_{345}$		${|{{\rm{\Psi }}}_{000}\rangle }_{11^{\prime} 2}{|{{\rm{\Psi }}}_{011}\rangle }_{345}$ or ${|{{\rm{\Psi }}}_{100}\rangle }_{11^{\prime} 2}{|{{\rm{\Psi }}}_{111}\rangle }_{345}$	
*XXX*1102 1								
*IIX*11′2 0	${|{{\rm{\Psi }}}_{001}\rangle }_{11^{\prime} 2}{|{{\rm{\Psi }}}_{000}\rangle }_{345}$ or ${|{{\rm{\Psi }}}_{101}\rangle }_{11^{\prime} 2}{|{{\rm{\Psi }}}_{100}\rangle }_{345}$		${|{{\rm{\Psi }}}_{001}\rangle }_{11^{\prime} 2}{|{{\rm{\Psi }}}_{001}\rangle }_{345}$ or ${|{{\rm{\Psi }}}_{101}\rangle }_{11^{\prime} 2}{|{{\rm{\Psi }}}_{101}\rangle }_{345}$		${|{{\rm{\Psi }}}_{001}\rangle }_{11^{\prime} 2}{|{{\rm{\Psi }}}_{010}\rangle }_{345}$ or ${|{{\rm{\Psi }}}_{101}\rangle }_{11^{\prime} 2}{|{{\rm{\Psi }}}_{110}\rangle }_{345}$		${|{{\rm{\Psi }}}_{001}\rangle }_{11^{\prime} 2}{|{{\rm{\Psi }}}_{011}\rangle }_{345}$ or ${|{{\rm{\Psi }}}_{101}\rangle }_{11^{\prime} 2}{|{{\rm{\Psi }}}_{111}\rangle }_{345}$	
*XXI*1102 0								
*IXI*11′2 0	${|{{\rm{\Psi }}}_{010}\rangle }_{11^{\prime} 2}{|{{\rm{\Psi }}}_{000}\rangle }_{345}$ or ${|{{\rm{\Psi }}}_{110}\rangle }_{11^{\prime} 2}{|{{\rm{\Psi }}}_{100}\rangle }_{345}$		${|{{\rm{\Psi }}}_{010}\rangle }_{11^{\prime} 2}{|{{\rm{\Psi }}}_{001}\rangle }_{345}$ or ${|{{\rm{\Psi }}}_{110}\rangle }_{11^{\prime} 2}{|{{\rm{\Psi }}}_{101}\rangle }_{345}$		${|{{\rm{\Psi }}}_{010}\rangle }_{11^{\prime} 2}{|{{\rm{\Psi }}}_{010}\rangle }_{345}$ or ${|{{\rm{\Psi }}}_{110}\rangle }_{11^{\prime} 2}{|{{\rm{\Psi }}}_{110}\rangle }_{345}$		${|{{\rm{\Psi }}}_{010}\rangle }_{11^{\prime} 2}{|{{\rm{\Psi }}}_{011}\rangle }_{345}$ or ${|{{\rm{\Psi }}}_{110}\rangle }_{11^{\prime} 2}{|{{\rm{\Psi }}}_{111}\rangle }_{345}$	
*XIX*1102 0								
*IXX*11′2 0	${|{{\rm{\Psi }}}_{011}\rangle }_{11^{\prime} 2}{|{{\rm{\Psi }}}_{000}\rangle }_{345}$ or ${|{{\rm{\Psi }}}_{111}\rangle }_{11^{\prime} 2}{|{{\rm{\Psi }}}_{100}\rangle }_{345}$		${|{{\rm{\Psi }}}_{011}\rangle }_{11^{\prime} 2}{|{{\rm{\Psi }}}_{001}\rangle }_{345}$ or ${|{{\rm{\Psi }}}_{111}\rangle }_{11^{\prime} 2}{|{{\rm{\Psi }}}_{101}\rangle }_{345}$		${|{{\rm{\Psi }}}_{011}\rangle }_{11^{\prime} 2}{|{{\rm{\Psi }}}_{010}\rangle }_{345}$ or ${|{{\rm{\Psi }}}_{111}\rangle }_{11^{\prime} 2}{|{{\rm{\Psi }}}_{110}\rangle }_{345}$		${|{{\rm{\Psi }}}_{011}\rangle }_{11^{\prime} 2}{|{{\rm{\Psi }}}_{011}\rangle }_{345}$ or ${|{{\rm{\Psi }}}_{111}\rangle }_{11^{\prime} 2}{|{{\rm{\Psi }}}_{111}\rangle }_{345}$	
*XII*11′2 1

**Figure 2:**
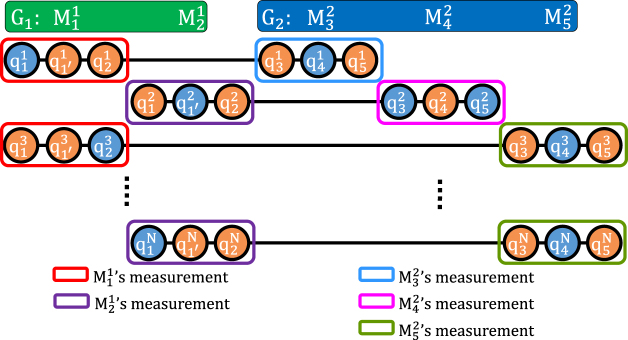
The final states of two group example.

### The proposed multi-group DGMQKA protocol

The basic idea of the two-group DGMQKA protocol can be generalized to multi-group cases in the same way. This means that there are *n* arbitrary groups, *G*_1_, *G*_2_, …, *G*_*n*_. and *m* arbitrary members, ${M}_{1}^{1}$, ${M}_{2}^{1}$, ${M}_{3}^{1}$, …, ${M}_{m}^{n}$. And every group will agree their group key. The multi-group case contains five steps as follows:

1. ${M}_{1}^{1}$ prepares an ordered sequence of *m*-qubit entangled state^21^, $\frac{1}{\sqrt{2}}{\mathrm{(000}\ldots 0+111\ldots \mathrm{1)}}_{123\ldots m}$. This sequence is composed of *N m*-qubit entangled states. Then ${M}_{1}^{1}$ splits it into *m* sequences, *S*_1_, *S*_2_,…, *S*_*m*_. After ${M}_{1}^{1}$ randomly inserts detection qubits^62^ into these sequences, with the exception of *S*_1_, ${M}_{1}^{1}$ sends these qubit sequences with detection qubits^62^ to all other members, respectively.
2. ${M}_{1}^{1}$ announces the positions and states of the detection qubits^62^ after ${M}_{2}^{1}$, …, ${M}_{m}^{n}$ receive *S*_2_, …, *S*_*m*_ with detection qubits^62^. And ${M}_{2}^{1}$, …, ${M}_{m}^{n}$ use the corresponding bases to measure these detection qubits^62^. If the error rate is higher than the threshold, this communication should be aborted; otherwise, they go to the next step.
3. After all groups go through steps as Step3 of “The proposed two-group DGMQKA protocol” section 0, the number of entangled qubits satisfies $\lceil \frac{P+1}{2}\rceil +\lceil \frac{P-1}{2}\rceil$, where *P* are the number of group members. Then, all members add their own idea for a key by performing operations on qubits in hand. The operation rule is that all members are able to perform one of two operations {*I*, *X*} on their qubits sequences. The two operations, *I* and *X*, represent that key is “0” or “1”. Then, every member will be the group leader in turns who measures the entangled states and announces the measurement results.
4. All members randomly insert the detection qubits^62^ as ${M}_{1}^{1}$ did in step 1 after the self key encryption. They send their qubit sequences with detection qubits^62^ to their group leader. After all group leaders receive the qubit sequences with detection qubits^62^ from members, they announce the positions and states of detection qubits^62^ for the channel checking, and check the error rate. They abort this communication if the error rate is higher than threshold; otherwise, they go to the next step.
5. All group leaders perform the GHZ measurement and announce the measurement results. After the measurement results are announced, all members can distinguish the operations of each group member and generate the group key.

### Key generation

Once the measurement results are announced, all group members can utilize the measurement result and their own operation to deduce operations performed by other members in same group. Because each member just performs the operations on the qubit on hand, each can determine one bit of the group key in a key agreement. The *I* operation indicates that the key is “0”; otherwise, the key is “1”. The final key appears when all the operation information turns to key information and utilize the XOR operation to transform all the operation results to one bit information. Thus, the final key is determined by the operations performed by all the group members.

For example, ${M}_{1}^{1}$ in *G*_1_ announces the measurement result which is ${|{{\rm{\psi }}}_{011}\rangle }_{11^{\prime} 2}$ ⇒ 011_11′2_, and ${M}_{1}^{1}$ and ${M}_{2}^{1}$ can utilize it and their own operations to deduce that the operation combination is *XII*_11′2_. However, the operation combination is in two possible measurement results. According to Table 1, find in same measurement results, have two possible operation combination here. Thus, every member needs to compare his/her operation with the measurement result. When the measurement result is different from the operations, it is flipped. That is to say, if the measurement result of the first qubit is 1, it will be turned into 0. Here, because the result of first qubit is 0_1_, it is still ${|011\rangle }_{11^{\prime} 2}$. However, if ${M}_{1}^{1}$ operation is *XI*_11′_(10). Because it is different from the measurement result of qubit 1 and 1’, ${|01\rangle }_{11^{\prime} }$, the measurement result is flipped to ${|100\rangle }_{11^{\prime} 2}$. Finally, ${M}_{1}^{1}$ obtains the group key which is 1⊕0⊕0 = 1. Following the same steps, ${M}_{2}^{1}$ can also get the group key “1”.

Here, we explain the condition of the number of entangled qubits: $\lceil \frac{P+1}{2}\rceil +\lceil \frac{P-1}{2}\rceil$, where *P* is the number of group participants. The aim is mainly to solve the same XOR result problem, which anyone can estimate the key, when the number of participants is even. So, all the group leaders decide whether the number of participants is even or odd, and then turn the number of entangled qubits into odd numbers. Thus, when *P* is 2, 3, 4, 5, …, *K*, the number of group entangled qubits have to be $\mathrm{3,}\,\mathrm{3,}\,\mathrm{5,}\,\mathrm{5,}\,\ldots ,\,\lceil \frac{K+1}{2}\rceil +\lceil \frac{K-1}{2}\rceil$. This proposed protocol will be problematic without the above condition. For example, there are two participants, *A* and *B*, in same group. If group leader *A* announces the measurement result is $|{\varphi }^{+}\rangle =\frac{1}{\sqrt{2}}{(|00\rangle +|11\rangle )}_{AB}$, the possible operation combinations which they perform are *II*(00) or *XX*(11). If they use the method of key generation without the above condition, they will obtain same key after XOR operation whatever they deduce. Therefore, as long as they announce the measurement results, all the people including non-participants and other members of groups can know the key. This problem always happens when the number of participants is even, and this is why this protocol needs the condition. To solve above problem, group leader *A* just adds a entangled qubit ${|0\rangle }_{A^{\prime} }$ into initial state $\frac{1}{\sqrt{2}}{(|00\rangle +|11\rangle )}_{AB}$ to $\frac{1}{\sqrt{2}}{(|000\rangle +|111\rangle )}_{AA^{\prime} B}$ and performs the GHZ measurement. After the announcement, we can find that the two possible operation combinations which they perform are *III*(000) or *XXX*(111). Besides, we also observe that the key generation is different from two possible operation combinations: *III*(000) is 0 after 0 ⊕ 0 ⊕ 0 = 0 and *XXX*(111) is 1 after 1 ⊕ 1 ⊕ 1 = 1. Therefore, only group members can use the measurement result and operations to generate the key; the others cannot.

In Zeng *et al*.^61^, the operation rule is that all participants perform one of two operations *I*, *X*, with the exception of one participant, who performs one of four operations *I*, *X*, *Y*, *Z*. Group members must take turns fulfilling this function. The key generating methods are different according to the number of participants that are even or odd. However, the operation rule of the proposed protocol is that every participant performs one of two operations *I*, *X* and there is only one key generating method.

### Dynamic in protocol

To make the proposed protocol more flexible, dynamic properties are added to it. It is able to perform four distinct dynamic actions, namely, 1. join members, 2. combine two groups into one group, 3. revoke members and 4. divide one group into two groups, to be discussed.

#### A new member joins

The process is that configure initial quantum state to all members including new members again in order to generate a new group key. Suppose that there is a group, *G*_1_, composed of ${M}_{1}^{1}$, ${M}_{2}^{1}$ and ${M}_{3}^{1}$. A new member, ${M}_{4}^{1}$, wants to join *G*_1_ and generate new group key. This process consists of five steps:

1. After the previous key agreement, because the group leaders have to take turns, ${M}_{i}^{1}$(*i* = 1, 2, 3) obtain $\frac{N}{3}$ three-qubit entangled states (because every member needs to take turns as a leader to collect entangled states for performing GHZ measurement) respectively. Then they perform the corresponding operations on all three-qubit entangled states to turn them into initial states $\frac{1}{\sqrt{2}}{(|000\rangle +|111\rangle )}_{123}$. After that, the number of entangled qubit has to be $\lceil \frac{P+1}{2}\rceil +\lceil \frac{P-1}{2}\rceil$, where *P* is the number of group members. Because of a new member joining, this condition is not satisfied odd number of the entangled qubit, ${M}_{i}^{1}$ splits the (*N*)/(3) three-qubit entangled states into three sequences: ${S}_{1}^{i}$, ${S}_{2}^{i}$ and ${S}_{3}^{i}$. After that, taking one leader as an example, the others do the same thing: ${M}_{1}^{1}$ prepares two sequences: ${S}_{4}^{1}$, composed of (*N*)/(3) single qubits in ${|0\rangle }_{4}$, and ${S}_{5}^{1}$, composed of (*N*)/(3) single qubits in ${|0\rangle }_{5}$. Then ${M}_{1}^{1}$ performs *CNOT*_14_ on ${S}_{1}^{1}$ and ${S}_{4}^{1}$, and performs *CNOT*_15_ on ${S}_{1}^{1}$ and ${S}_{5}^{1}$. Therefore, every three-qubit entangled state will be turned into a five-qubit entangled state. Then ${M}_{1}^{1}$ renames his sequence from ${S}_{1}^{1},{S}_{2}^{1},\cdots ,{S}_{5}^{1}$ to ${S}_{1}^{1},{S}_{1^{\prime} }^{1},\cdots ,{S}_{4}^{1}$. After that, ${M}_{1}^{1}$ inserts detection qubits^62^ into every sequence except ${S}_{1}^{1}$ and ${S}_{1^{\prime} }^{1}$, and sends ${S}_{2}^{1}$, ${S}_{3}^{1}$ and ${S}_{4}^{1}$ with detection qubits^62^ to ${M}_{2}^{1}$, ${M}_{3}^{1}$ and ${M}_{4}^{1}$ respectively.
2. ${M}_{i}^{1}(i=1,2,\mathrm{3)}$ announce the positions and states of detection qubits^62^ after other members receive sequences with detection qubits^62^. And all members can use the corresponding bases to measure these detection qubits^62^. If the error rate is higher than the threshold, this communication should be aborted. Otherwise, they go to the next step.
3. The operation rule is that all members are able to perform one of two operations: {*I*, *X*} on their qubits sequences. The two operations, *I* and *X*, represent that the key is “0” or “1”. Then, every member will be the group leader in turn who measures the entangled states and announces the measurement results. Hence, the order of the group leader in *G*_1_ is: ${M}_{1}^{1}$, ${M}_{2}^{1}$, ${M}_{3}^{1}$, ${M}_{4}^{1}$,${M}_{1}^{1}$, ${M}_{2}^{1}$, ${M}_{3}^{1}$, ${M}_{4}^{1}$, …, ${M}_{4}^{1}$.
4. After the key encryption, all members randomly insert the detection qubits^62^ for channel checking. They send their qubit sequences to their group leader. When every group leader has received the qubit sequences with detection qubits^62^ from the members, they announce the positions and states of the detection qubits^62^ for the channel checking, and check the error rate. They abort this communication if the error rate is higher than the threshold; otherwise, they go to the next step.
5. All group leaders perform the GHZ measurement and announce the measurement results. All members can obtain the group key through the measurement results and their own operation information.

#### Combine two groups into one group

They exchange their old operations (key) information to each other in order to combine into one group. If three or more groups want to combine into one group, the process must be undergone in group pairs. Suppose that members of the *G*_1_, composed of ${M}_{1}^{1}$ and ${M}_{2}^{1}$, and *G*_2_, composed of ${M}_{3}^{2}$, ${M}_{4}^{2}$, and ${M}_{5}^{2}$, want to combine into one group. They must exchange operations information of the two groups. It requires all three-qubit entangled states which are held by every member respectively. The process involves six steps:

1. Take one member as an example, ${M}_{1}^{1}$ turns his all three-qubit entangled states into $\frac{1}{\sqrt{2}}{(|000\rangle +|111\rangle )}_{11^{\prime} 2}$. ${M}_{1}^{1}$ splits all three-qubit entangled states into three sequence ${S}_{1}^{1}$, ${S}_{1^{\prime} }^{1}$, and ${S}_{2}^{1}$. Then ${M}_{1}^{1}$ inserts detection qubits^62^ randomly into sequence ${S}_{2}^{1}$ and sends it to ${M}_{3}^{2}$.
2. After ${M}_{3}^{2}$ receives the sequence with detection qubits^62^, ${M}_{1}^{1}$ informs ${M}_{3}^{2}$ of the positions and states of the detection qubits^62^ for channel checking. They abort this communication if the error rate is higher than the threshold; otherwise, they go to the next step.
3. ${M}_{3}^{2}$ performs the operations which are the group keys at every qubit in the sequence. After performing operations on every qubit in the sequence, ${M}_{3}^{2}$ randomly inserts detection qubits^62^ into the sequence and send them with detection qubits^62^ to ${M}_{1}^{1}$.
4. After ${M}_{1}^{1}$ receives the ${S}_{2}^{1}$ with detection qubits^62^, ${M}_{3}^{2}$ announces the states and positions of detection qubits^62^ for channel checking, and they check the error rate. They abort this communication if the error rate is higher than threshold. Otherwise, they go to the next step.
5. After channel checking, ${M}_{1}^{1}$ performs operations on corresponding qubit 1 and 2 in every three-qubit entangled states. The operation on qubit 1 is the group key of *G*_1_ and the operation on qubit is one of two operations {*I*, *X*}. And the operation on qubit 1’ is the random operation from {*I*, *X*}. ${M}_{1}^{1}$ performs the GHZ measurement on those entangled states and announce the measurement results. Every member can obtain the new group key by own original group key and measurement results.
6. One member can generate new $\frac{N}{5}$ -bit key. After all members perform above all steps, they can generate new *N*-bit group key.

#### Revoke an old member

This is the process by which all members except an evicted member configure a fresh initial GHZ state for generating a new group key. Suppose that there is a group *G*_1_ which is composed of ${M}_{1}^{1}$, ${M}_{2}^{1}$, ${M}_{3}^{1}$ and ${M}_{4}^{1}$. ${M}_{1}^{1}$, ${M}_{2}^{1}$ and ${M}_{3}^{1}$ want to revoke an original member, ${M}_{4}^{1}$. The process for this involves five steps:

1. After the previous key agreement, because the group leaders have to take turns, ${M}_{i}^{1}$(*i* = 1, 2, 3, 4) obtain $\frac{N}{4}$ five-qubit entangled states, respectively. Then they perform corresponding operations on all five-qubit entangled states to turn them into initial state $\frac{1}{\sqrt{2}}{(|00000\rangle +|111111\rangle )}_{11^{\prime} 234}$. After that, the number of entangled qubit has to be $\lceil \frac{P+1}{2}\rceil +\lceil \frac{P-1}{2}\rceil$, where *P* is the number of group members. Because this odd condition is not satisfied, ${M}_{i}^{1}$ splits the $\frac{N}{4}$ five-qubit entangled states into five sequences: ${S}_{1}^{i}$, ${S}_{1^{\prime} }^{i}$, ${S}_{2}^{i}$, ${S}_{3}^{i}$ and ${S}_{4}^{i}$, and performs *CNOT*_11′_ on ${S}_{1}^{i}$ and ${S}_{1^{\prime} }^{i}$, and performs *CNOT*_14_ on ${S}_{1}^{i}$ and ${S}_{4}^{i}$. *N* five-qubit entangled states will be turned into *N* three-qubit entangled states. After that, taking one leader as an example, the others do the same thing: ${M}_{1}^{1}$ inserts the detection qubits^62^ into ${S}_{2}^{1}$ and ${S}_{3}^{1}$ and sends ${S}_{2}^{1}$ and ${S}_{3}^{1}$ with the detection qubits^62^ to ${M}_{2}^{1}$ and ${M}_{3}^{1}$, respectively.
2. ${M}_{i}^{1}$(*i* = 1, 2, 3) announces the positions and states of the detection qubits^62^ after other members receive sequences with detection qubits^62^. All the members use the corresponding bases to measure these detection qubits^62^. If the error rate is higher than the threshold, this communication should be aborted; otherwise, they go to the next step.
3. The operation rule is that all members are able to perform one of two operations {*I*, *X*} on their qubits sequences. The two operations, *I* and *X*, represent that key is “0” and “1”, respectively. Then, every member will be the group leader in turn who measures the entangled states and announces the measurement results. Hence, the order of group leader in *G*_1_ is ${M}_{1}^{1}$, ${M}_{2}^{1}$, ${M}_{3}^{1}$,${M}_{1}^{1}$, ${M}_{2}^{1}$, ${M}_{3}^{1}$, …, ${M}_{3}^{1}$.
4. After the key encryption, all members randomly insert the detection qubits^62^ for channel checking. They send their qubit sequences to their group leader. When every group leader has received the qubit sequences with detection qubits^62^ from members, they announce the positions and states of the detection qubits^62^ for the channel checking, and check the error rate. They abort this communication if the error rate is higher than the threshold; otherwise, they go to the next step.
5. All group leaders perform the GHZ measurement and announce the measurement results. All members can obtain the group key through the measurement results and their own operation information.

#### Divide one group into two groups

This process is similar to that for the proposed two-group DGMQKA protocol. Suppose that there is a group, *G*_1_, composed of ${M}_{1}^{1}$, ${M}_{2}^{1}$, ${M}_{3}^{1}$, ${M}_{4}^{1}$ and ${M}_{5}^{1}$. ${M}_{3}^{1}$, ${M}_{4}^{1}$ and ${M}_{5}^{1}$ want to leave *G*_1_ to form a new group *G*_2_, and the original *G*_1_ will be divided into two groups. The process involves five steps:

- After generating the group key, every member receives $\frac{N}{5}$ five-qubit entangled states respectively. Every member performs the corresponding operations on all $\frac{N}{5}$ five-qubit entangled state for turning all $\frac{N}{5}$ five-qubit entangled state into: $\frac{1}{\sqrt{2}}{(|00000\rangle +|11111\rangle )}_{12345}$. Every member splits them into five sequences and inserts detection qubits^62^ into every sequence except their own sequence. After that every member sends all the sequences with detection qubits^62^ to other members.
- Every member announces the positions and states of detection qubits^62^ after other members have received sequences with detection qubits^62^. All members are able to use the corresponding bases to measure these detection qubits^62^. If the error rate is higher than the threshold, this communication should be aborted; otherwise, they go to the next step.
- The number of members in *G*_1_ is 2, so the number of entangled qubits has to be 3. Therefore, there is a member in *G*_1_ to prepare a sequence which is composed of N/5 single qubits in $|0\rangle$. Here, suppose that ${M}_{1}^{1}$ prepares a sequence *S*_1′_ composed of N/5 single qubits in ${|0\rangle }_{1^{\prime} }$. Then, ${M}_{1}^{1}$ performs the *CNOT*_11′_ operation on *S*_1_ and *S*_1′_, where each qubit in *S*_1_ is a control bit and the corresponding each qubit in *S*_1′_ is a target bit. Every five-qubit entangled state: $\frac{1}{\sqrt{2}}{(|00000\rangle +|11111\rangle )}_{12345}$ will be turned into six-qubit entangled state: $\frac{1}{\sqrt{2}}{(|000000\rangle +|111111\rangle )}_{11^{\prime} 2345}$. The number of members in *G*_2_ is 3, so the number of entangled qubits have to be 3 and this condition is satisfied. After all the groups have gone through the above steps, all members add their own idea for a key by performing operations on qubits in hand. The operation rule is that all members are able to perform one of two operations {*I*, *X*} on their qubits sequences. The two operations: *I* and *X*, represent that key is “0” and “1”, respectively. Then, every member will be the group leader in turns who measures the entangled states and announces the measurement results. Hence, the order of group leader in *G*_1_ is ${M}_{1}^{1}$, ${M}_{2}^{1}$, ${M}_{1}^{1}$, …, ${M}_{2}^{1}$ and the order of group leader in *G*_2_ is ${M}_{3}^{2}$, ${M}_{4}^{2}$, ${M}_{5}^{2}$, …, ${M}_{5}^{2}$.
- After the key encryption, all members randomly insert the detection qubits^62^ for channel checking. They send their qubit sequences to their group leader. When every group leader receives the qubit sequences with detection qubits^62^ from members, they announce the positions and states of the detection qubits^62^ for the channel checking, and check the error rate. They abort this communication if the error rate is higher than the threshold; otherwise, they go to the next step.
- Finally, the two group leaders perform GHZ measurement and announce the measurement results for generating a group key. As a result, the members of the two groups have their respective group keys and one group has successfully been divided into two groups.

### Security analysis

This section will discuss External and Internal attacks. An external attack is any situation in which an eavesdropper wants to obtain the key agreement results. An internal attack is a situation in which any group member is able to determine the key agreement results.

#### External attack

In the following discussion, an eavesdropper, Eve, wants to extract the secret key without being detected. Secret messages can only be encrypted after the key is generated. This means that secret messages are not leaked if Eve is detected trying to steal the key. Therefore, this section will only discusses the situation in which Eve steals the key and has not been detected. Four common attack strategies can be used to achieve this goal: an intercept-and-resend attack, a control-not attack, an entangling attack, and a Trojan horse attack.

The intercept-resend attack is addressed first. In the proposed protocol, the sender will insert detection qubits^62^ into a qubit sequence with random states and positions in every qubit resource transmission. If Eve intercepts all qubits and measures them to try to get the operations performed by group members, she may change the states of the detection qubits^62^ because she doesn’t know the detection qubits^62^, correct states and positions. She will be detected by the $\frac{1}{4}$ probability with single qubit^1,62^. There is a ${(1-\frac{3}{4})}^{n}$ probability that Eve will not be detected with a continuous n single detection qubit.

Next, the control-not attack is addressed. Eve uses a control-not gate to steal the information of operations performed by group members^63^. Suppose that there is a group *G*_1_ composed of two members, ${M}_{1}^{1}$ and ${M}_{2}^{1}$. ${M}_{1}^{1}$ prepares a three-qubit entangled state^21^, $\frac{1}{\sqrt{2}}{(|000\rangle +|111\rangle )}_{{11}^{^{\prime} }2}$. Before ${M}_{2}^{1}$ receives qubit 2 from ${M}_{1}^{1}$, Eve performs *CNOT*_2,*E*_ on qubit 2 and qubit E, which is ${|0\rangle }_{{E}}$, where qubit 2 is a control bit and qubit E is a target bit. Then, Eve sends qubit 2 to ${M}_{2}^{1}$. After ${M}_{2}^{1}$ performs the operation for generating a group key, Eve performs *CNOT*_D,*E*_ again. Then, Eve performs Z-basis measurement on qubit E. If the measurement result of qubit E is ${|0\rangle }_{{E}}$, it means that the operation of ${M}_{2}^{1}$ is *I*_2_; otherwise, the operation of ${M}_{2}^{1}$ is *X*_2_. However, Eve cannot know what qubits are detection qubits^62^ for channel checking, and she may change the states of these detection qubits^62^ and thus be detected. Eve performs *CNOT*_D,*E*_, where the control bit is detection qubit $D\,{|+\rangle }_{D}$ and target bit is 0_*E*_ as (1). After this, qubit D will be entangled with qubit E, such that the state qubit D may be measured to $\,{|-\rangle }_{D}$ in X-basis. If the measurement result of qubit D is $\,{|-\rangle }_{D}$, Eve can be detected. However, if the state of detection qubit D is $\,{|0\rangle }_{D}$ or $\,{|1\rangle }_{D}$, Eve cannot be detected because qubit D isn’t entangled with qubit E. The detection probability is $\frac{1}{4}$ with a single qubit for channel checking.

1 $$\begin{array}{c}{|+0\rangle }_{DE}=\frac{1}{\sqrt{2}}{(|00\rangle +|10\rangle )}_{DE}\mathop{\Rightarrow }\limits^{CNO{T}_{D,E}}\frac{1}{\sqrt{2}}{(|00\rangle +|11\rangle )}_{DE}=\frac{1}{2}{(|++\rangle +|--\rangle )}_{DE}\end{array}$$

Thirdly, entangling attack is discussed. Suppose Eve intercepts a sequence *S*_*i*_(*I* = 2, 3, ..., *n*), where *n* is the number of participants, and performs a unitary operation *U* on the intercepted qubits to entangle an ancillary qubit *E* prepared in advance. The unitary operation *U* can be defined by the following equations:

2 $$\begin{array}{rcl}U(|0\rangle |E\rangle ) & = & a|0\rangle |{e}_{00}\rangle +b|1\rangle |{e}_{01}\rangle ,\,U(|1\rangle |E\rangle )=c|0\rangle |{e}_{10}\rangle +d|1\rangle |{e}_{11}\rangle ,\\ U(|+\rangle |E\rangle ) & = & \frac{1}{\sqrt{2}}(a|0\rangle |{e}_{00}\rangle +b|1\rangle |{e}_{01}\rangle +c|0\rangle |{e}_{10}\rangle +d|1\rangle |{e}_{11}\rangle )\\  & = & \frac{1}{2}(|+\rangle (a|{e}_{00}\rangle +b|{e}_{01}\rangle +c|{e}_{10}\rangle +d|{e}_{11}\rangle ))\\  &  & +\frac{1}{2}(|-\rangle (a|{e}_{00}\rangle -b|{e}_{01}\rangle +c|{e}_{10}\rangle -d|{e}_{11}\rangle )),\\  & = & \frac{1}{\sqrt{2}}(a|0\rangle |{e}_{00}\rangle +b|1\rangle |{e}_{01}\rangle -c|0\rangle |{e}_{10}\rangle -d|1\rangle |{e}_{11}\rangle )\\ U(|-\rangle |E\rangle ) & = & \frac{1}{2}(|+\rangle (a|{e}_{00}\rangle +b|{e}_{01}\rangle -c|{e}_{10}\rangle -d|{e}_{11}\rangle ))\\  &  & +\frac{1}{2}(|-\rangle (a|{e}_{00}\rangle -b|{e}_{01}\rangle -c|{e}_{10}\rangle +d|{e}_{11}\rangle ))\end{array}$$

where $|{e}_{00}\rangle$, $|{e}_{01}\rangle$, $|{e}_{10}\rangle$ and $|{e}_{11}\rangle$ are four states decided by the unitary operation *U*, |*a*|^2^ + |*b*|^2^ = 1 and |*c*|^2^ + |*d*|^2^ = 1. If Eve does not want to be detected in the channel checking, she cannot change the state of the qubits in *S*_*i*_. Therefore, the operation *U* must satisfy *a* = *d* = 1, *b* = *c* = 0 and $\,|{e}_{00}\rangle$ = $\,|{e}_{11}\rangle$. However, Eve cannot distinguish between $\,|{e}_{00}\rangle$ and $\,|{e}_{11}\rangle$, and this means that Eve cannot distinguish among $\,|0\rangle$, $\,|1\rangle$, $\,|+\rangle$ and $\,|-\rangle$. As a result, Eve cannot obtain any useful information, and the entangling attack is unsuccessful for the proposed protocol.

Finally, let us discuss the Trojan horse attack. If the qubit is a photon, there are two kind of Trojan horse attacks: delay-photon^64^ and the invisible photon^65^ attack. In order to handle the problems, the specific detecting devices are used, such as photon number splitter (PNS) and wavelength filter. For the delay-photon attack, the PNS can be used to count the number of photons for detecting whether excess exists in the signal transmission. As for the invisible attack, a wavelength filter (WF) can be added before the receiver’s device which can leach out the spy photons and thus prevent the invisible photon attack.

In our protocol, the qubit sequence are always inserted into detection qubits^62^ in every transmission. Our protocol can defend above three common external attack through the detection qubits^62^. Moreover, the specific devices, which are PNS and WF, are added for defeating the Trojan horse attack. In addition, in dynamic, join a new member, combine two groups into one group, revoke an old member, and divide one group into two groups, it is also secure because of detection qubits^62^ in every transmission. Therefore, our protocol won’t be attacked by eavesdropper whether it is in dynamic or not.

#### Internal attack

In this proposed protocol, after the measurement results are announced, all group members know the group key. As a result, the member who announces the measurement results is every important. Take the above two-group case for example; if the group operations of *G*_2_ are *III*_345_, the measurement results should be $|{{\rm{\Psi }}}_{000}\rangle$ or $|{{\rm{\Psi }}}_{100}\rangle$. Group leader, ${M}_{3}^{2}$ who announces the measurement results, would like to decide the group key by announcing measurement results which are different from the original. In addition, the group key must be generated afresh when a new member is added to a group, when two groups combine into one group, when a member is revoked from a group, and when one group is split into two groups. If the group leaders are dishonest, the group key also can be controlled through announcing different measurement. Therefore, the group leader has to be by turns. It can sure that no one can control the whole key string.

### Consumption comparison

Three standards of comparison are use in this study, namely, “number of transmissions”, “number of qubit measurement”, and “number of qubit for channel checking”. The proposed protocol is compared with 8 current MQKA protocols, namely “Shi and Zhong^53^”, “Liu *et al*.^54^”, “Shukla *et al*.^55^” (Zhu *et al*.^56^ pointed out Shukla *et al*.’s protocol is insecurity and modified it with additional classical message, but the consumption is almost the same as the original one, so we count it into this section), “Sun *et al*. 1^57^”, “Sun *et al*. 2^58^”, “Huang *et al*.^59^”, “Cao and Mao^60^” and “Zeng *et al*.^61^”. Although the proposed protocol can be used in two groups or more, it will be compared with other protocols using only one group. Table 2 shows the detailed consumption comparison between these 8 MQKA protocols and ours. The consumption analysis will be described as follows:

**Table 2:** Consumption comparison.

	Transmission	Measurement	Qubit for channel checking
Shi^53^	*N* ^2^	2*N*^2^	20*N*^2^
Liu^54^	2(*N*^2^ − *N*)	2(*N*^2^ − *N*)	40(*N*^2^ − *N*)
Shukla^55^	2*N*^2^	4*N*	40*N*^2^
Sun1^57^	*N* ^2^	4*N*	20*N*^2^
Sun2^58^	2(*N*^2^ + *N*)	6*N*	40(*N*^2^ + *N*)
Huang^59^	2*N*^2^	2*N*	40*N*^2^
Cao^60^	2*N*^2^	2*N*	40*N*^2^
Zeng^61^	4(*N* − 1)	2*N*	80(*N* − 1)
Our	4(*N* − 1)	2*N*	80(*N* − 1)

#### Number of transmission

**Figure 3:**
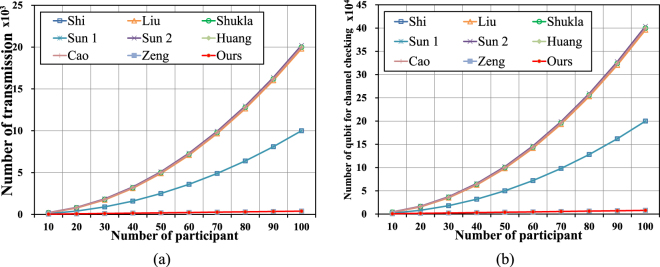
(**a**) Comparison of number of transmission: ours and references^53–55,57–61^, (**b**) Comparison of number of qubit for channel checking: ours and references^53–55,57–61^.

Using the same key length key agreement, the proposed protocol is compared with 8 current MQKA protocols in terms of number of transmissions. Each qubit is counted as the number of transmissions from all participants, with the exception of qubits for channel checking. Here, *N* is the number of participants. According to Table 2, the number of transmissions of all current MQKA protocols is *N*^3^ except^61^, because of unicast transmission. The performance of the proposed protocol is same as Zeng *et al*.’s protocol^61^, because those two protocols are based on the MQSDC protocol^25^. However, the proposed protocol is more flexible. Therefore, the numbers of transmissions of these current MQKA protocols are higher than that of the proposed protocol. The total comparison is shown in Fig. 3.

#### Number of qubit measurement

**Figure 4:**
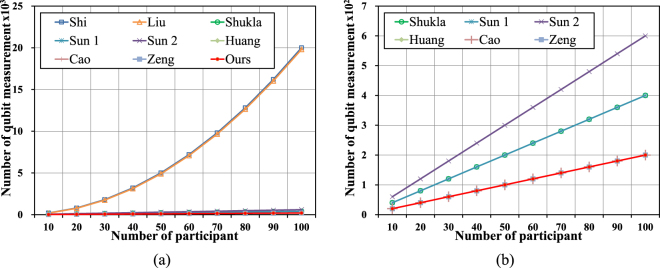
Comparison of number of qubit measurement: (**a**) Comparisons between our protocol and references^53–55,57–61^, (**b**) Comparisons between our protocol and references^55,57–61^ (enlarged Fig. 4a).

In this section, the number of qubits measurement is discussed. Different quantum states are used in the current MQKA protocol, such as single qubit, Bell states, GHZ states, four-qubit cluster states and six-qubit states. Current MQKA protocols are able to finish the agreement process after the measurement. The greater the number of qubits measured, the higher the cost. Assume that same length key is agreed in every MQKA protocol, and one qubit measurement will be counted once^53^. takes a large number of qubits because it has to run Bell state measurements in every transmission^54^. must prepare many single qubits and requires a large number of qubits. Others^55,57–61^ and the proposed protocol require fewer qubits because these protocols only measure qubits in the last transmission. The proposed protocol only needs the GHZ measurement once, the same as Zeng *et al*.’s protocol^61^, because they are based on MQSDC^25^. Details are shown in Fig. 4.

#### Number of qubit for channel checking

The number of qubits used for channel checking should be discussed with sequence transmission. These sequences are inserted into the qubits for channel checking. In this section, sequences are composed of 200 qubits. There are 20 qubits for channel checking in a sequence. The probability of an eavesdropper being detected is $1-{(\frac{3}{4})}^{20}\approx 0.997$. Without losing fairness, each protocol agrees on a 180*N* bit key, where *N* is the number of participants. Because of different transmission methods, those protocols^53–55,57–60^ using unicast transmission require more qubits for channel checking than in Zeng *et al*.^61^ and the proposed protocol using multicast transmission. Therefore, our proposed protocol is more flexible than Zeng *et al*.’s protocol^61^. Details are given in Fig. 3.

### Implementation discussion

The proposed protocol mainly uses GHZ states. The entangled state, however, is difficult to maintain on a noisy channel, especially in dealing with a multiple entangled state. In addition, there are two important issues: absorption and decoherence. First is the absorption issue: the transmission distance of sender and receiver is also a challenging issue because the qubit is easily absorbed by the channel. Second is the decoherence issue: the qubit state easily interacts with the environment, leading to the state being changed because the qubit is too small to be disturbed. Many researchers have tried to solve these issues by the quantum repeater with the techniques of entanglement purification and concentration. Bennett *et al*. first proposed two protocols for entanglement purification^66^ and concentration^67^, where the purification extracts one of the four Bell states for mixed states and concentration adjusts the amplitude to the maximally entangled states for pure state. The entanglement purification and concentration can give ideal entangled states to the agents. Briegel *et al*.^68^ proposed a quantum repeater protocol by using entanglement swapping to exchange the entangled chain repeatedly till to create a quantum channel between the sender and receiver. So far, many protocols on purification^69–72^, concentration^73–77^ and repeater have been implemented and experimented on to create a pure Bell state. Those techniques caused the practicality of quantum repeater^78,79^ to become mature. Furthermore, more types of entangled states concerning purification and concentration have been subject to experiments and applications, such as multipartite entanglement purification^71^, concentration in graphene^77^, GHZ^80^, cluster^81^ state, N-particle W state^76^, and an application for quantum blind computation^72^.

The proposed protocol can use only the concept of Briegel *et al*.’s quantum repeater^68^ for implementation, which distributes only two entangled states for the sender and receiver, and can be currently implemented. Using the technique of Chou *et al*.’s MQSS^40^ protocol, the sender can distribute the Bell state by quantum repeater and perform GHZ measurement based on the qubits he holds. The multiple Bell state entangled chain will become a GHZ state; then the GHZ distribution can be completed. Another advantage of the proposed protocol is that it can work well with same initial state, i.e. they can distribute the GHZ state in the beginning when they get together. This advantage is very suitable for a wireless sensor network because these sensors get together in the beginning. After this, during the key generation stage, those agents can perform nonlocal controlled-gate^82–84^ operations to accomplish the GHZ measurement without GHZ state transmission, by only consuming a Bell state between each controlled and target bit. In the future, a quantum internet^85^ will become more and more feasible, and save additional cost for the consumption of a quantum repeater and nonlocal operations.

## Conclusion

This study successfully proposes a novel dynamic group MQKA protocol. The protocol is the first MQKA with dynamic group properties. Thus, the proposed protocols are fundamentally different from all the existing MQKA protocols. Because of the multicast transmission method, the proposed protocol is more effective than other MQKA protocols. In addition, its dynamic nature makes the protocol more flexible. On the other hand, the proposed protocol is also helpful for research of QSS because the two complement each other. In the security analysis section of this paper, the proposed protocol was able to defend external eavesdropper attacks and internal malicious participant attacks. In the consumption comparison section, the performance of the proposed protocol was shown to be superior to the compared MQKA protocols^53–55,57,58,60,61^.

### Data availability

No datasets were generated or analysed during the current study.
